# Rhinitis, sinusitis and ocular disease – 2086. Nox4 mediates hypoxia-stimulated myofibroblast differentiation in nasal polyp-derived fibroblasts

**DOI:** 10.1186/1939-4551-6-S1-P165

**Published:** 2013-04-23

**Authors:** Heung-Man Lee, Sung-Moon Hong, Jung-Sun Cho

**Affiliations:** 1 Otorhinolaryngology, Korea University College of Medicine, Guro Hospital, Seoul, South Korea; 2 Otorhinolaryngology, Korea University Hospital, South Korea

## Background

Chronic hypoxia is associated with remodeling in various organs. Reactive oxygen species (ROS) derived from NADPH oxidases (Nox), and TGF-beta1 have been implicated in the pathogenesis of hypoxia-induced remodeling. The aims of this study were to determine in hypoxia-stimulated nasal polyp-derived fibroblasts (NPDF) the effect of hypoxia on the differentiation of myofibroblasts, the role of ROS, the major Nox homolog mediating myofibroblast differentiation, and the role of TGF-beta1.

## Methods

Eight primary cultures of NPDF were established from nasal polyps, which were incubated under hypoxic conditions. Reverse transcription polymerase chain reaction for *alpha-SMA*, *Nox1*, *Nox3*, *Nox4*, *Nox5*, and *fibronectin* mRNA was performed. Western blotting for alpha-SMA and fibronectin was done. ROS production was detected using a fluorometer. NPDF were pretreated with ROS scavengers and transfected with siNox4. The TGF-beta1 protein level was measured by ELISA. The effect of treatment with TGF-beta1 type I tyrosine kinase inhibitor SB431542 on myofibroblast differentiation was ob-served.

## Results

Hypoxic stimulation of NPDF significantly increased alpha-SMA and fibronectin mRNA and protein expression. ROS production was increased by hypoxia, and ROS scavengers inhibited myofibroblast differentiation. Nox4 mRNA was the only Nox homolog increased by hypoxia. Transfection with siNox4 inhibited myofibroblast differentiation. TGF-beta1 was secreted endogenously by hypoxic NPDF. SB431542 significantly inhibited myofibroblast differentiation.

## Conclusions

Hypoxia induces myofibroblast differentiation of NPDF through a signaling pathway involving Nox4-dependent ROS generation and TGF-beta1. Therapies targetingNox4 may be effective against remodeling of nasal polyps.

